# Setting the stage: The initial immune response to blood-stage parasites

**DOI:** 10.1080/21505594.2019.1708053

**Published:** 2020-01-03

**Authors:** Allison N. Bucşan, Kim C. Williamson

**Affiliations:** Department of Microbiology and Immunology, Uniformed Services University of the Health Sciences, Bethesda, MD, USA

**Keywords:** (5–10): Malaria, *Plasmodium falciparum*, monocytes, dendritic cells, gamma-delta T cells, NK cells

## Abstract

Individuals growing up in malaria endemic areas gradually develop protection against clinical malaria and passive transfer experiments in humans have demonstrated that this protection is mediated in part by protective antibodies. However, neither the target antigens, specific effector mechanisms, nor the role of continual parasite exposure have been elucidated, which complicates vaccine development. Progress has been made in defining the innate signaling pathways activated by parasite components, including DNA, RNA, hemozoin, and phospholipids, which initiate the immune response and will be the focus of this review. The challenge that remains within the field is to understand the role of these early responses in the development of protective adaptive responses that clear iRBC and block merozoite invasion so that optimal vaccines and therapeutics may be produced.

## Graphical Abstract

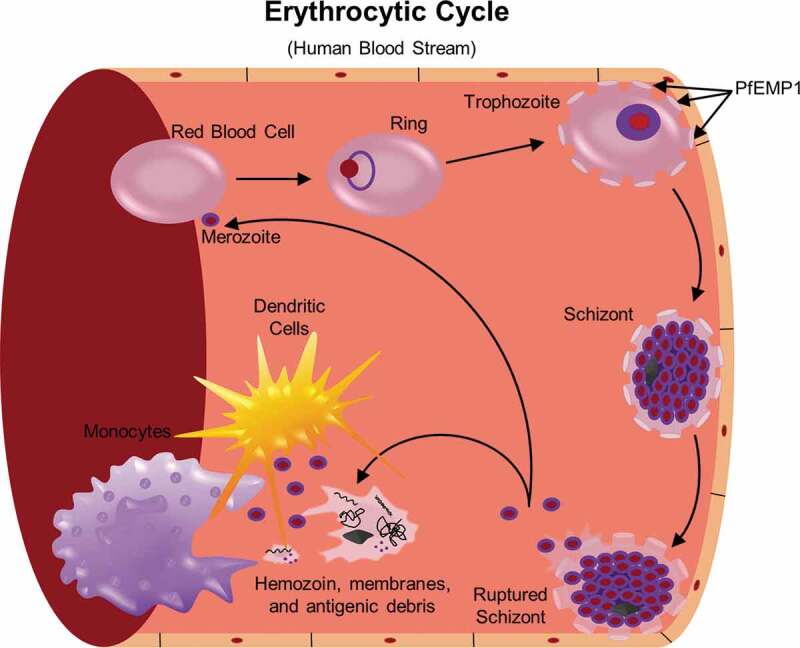

## Introduction

Malaria continues to be one of the most prevalent infectious diseases, causing 219 million cases in 2017 in 87 countries [1]. After repeated bouts of malaria, individuals living in endemic regions gradually gain resistance against severe disease. Consequently, 61% of all deaths due to malaria occurred in children under 5 years old [1]. Although, natural immunity is known to be acquired gradually through repeated exposures during childhood in malaria endemic areas, factors that influence the development of a protective immune response are poorly understood. Here we will review the immune responses that occur during the initial phases of *Plasmodium* infection that may influence the development of immunity to blood-stage parasites with a focus on *Plasmodium falciparum*, the parasite species responsible for the most severe form of human malaria.

## Host response to erythrocytic asexual stage *P. falciparum*

The host immune response is traditionally divided into 2 arms, the innate and the adaptive, based on the types of antigen receptors utilized. Cells involved in the innate response express germline encoded receptors that do not vary from cell to cell. These cells, which include neutrophils, monocytes, macrophages, and NK cells, can respond immediately when triggered by antigens to provide a first line of defense and initiate the subsequent adaptive immune response by secreting chemokines and cytokines to recruit and activate additional cells. In contrast, cells involved in the adaptive response express receptors formed by RAG (recombination-activating gene)-mediated recombination of germline exons that occurs during T and B cell development. Consequently, each new T and B cell produced by the host expresses a distinct receptor with different binding properties that allows each cell to recognize a unique antigen [2]. This process provides a diverse repertoire of receptors in circulation at any given time. However, to generate an effective response, the few T or B cells expressing receptors that recognize a specific antigen first have to be stimulated to proliferate to amplify the population expressing those antigen-specific receptors. This expansion of reactive T and B cells takes time and, therefore, to control the initial exposure to a virulent pathogen like *P. falciparum*, either maternal antibodies, in the case of infants born in endemic areas, or effective chemotherapy are often needed. The importance of effective chemotherapy to facilitate parasite clearance, even with an intact immune response, was clearly demonstrated by the marked increase in malaria mortality observed with the spread of chloroquine-resistant parasites in the 1990s [3]. A better understanding of the dynamic host-parasite interaction is needed to direct the development of more effective interventions such as long-lasting vaccines.

## The *P. falciparum* life cycle – a challenge for immune recognition

**Figure 1.**
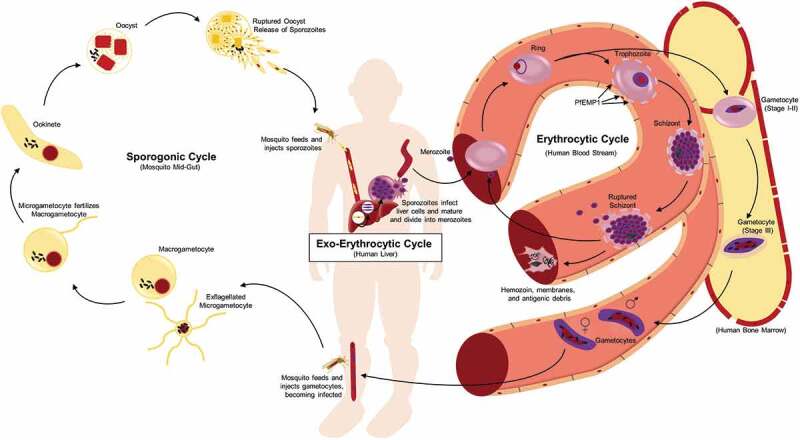
**Life cycle of *P. falciparum***. Infection of the human host occurs when a female *Anopheles* mosquito bites and injects *P. falcipraum* sporozoites from their salivary glands into ahost capillary during a blood meal. Sporozoites that enter the bloodstream travel to the liver and invade hepatocytes. Over the course of 7 days, a single sporozoite undergoes asexual reproduction within a hepatocyte to produce ~40,000 merozoites that are released into the bloodstream when the hepatocyte ruptures. The released merozoites invade erythrocytes, beginning the 48 hr erythrocytic life cycle as ring stage parasites. During maturation to a trophozoite, the parasites modify the erythrocyte surface by forming knobs containing PfEMP1 proteins that adhere to the microvasculature and prevent parasite clearance by the spleen. The parasite remains sequestered as it undergoes 4–5 rounds of asexual reproduction, producing a schizont containing 16–32 merozoites that are released during schizont rupture along with hemozoin, membranes, and antigenic debris that can stimulate early innate immunity. A subset of intraerythrocytic parasites undergo sexual differentiation and develop for 10–12 days within the bone marrow into either a male or a female gametocyte. Mature stage V gametocytes re-enter the circulation and can be taken up by a female mosquito to propagate the infection cycle. Within the mosquito midgut, these male and female gametocytes are stimulated immediately to form microgametes and macrogametes, respectively, which fertilize. Over the next 24 hr, the zygote develops into an ookinete, migrates across the midgut epithelium and becomes an oocyst that in 2–3 weeks can produce thousands of sporozoites. The sporozoites are released upon oocyst rupture and migrate to the mosquito salivary glands, ready to begin the cycle in a new human host.

In addition to the time needed for the development of an adaptive immune response, stark differences in protein expression and tissue tropism of the *Plasmodium* parasite through its life cycle complicate immune recognition and elimination. Infection is initiated when a female *Anopheles* mosquito carrying *Plasmodium* sporozoites in their salivary glands takes a human blood meal and introduces sporozoites into the skin and capillaries (Figure 1). If sporozoites do not successfully migrate to a capillary, they die within hours and can be taken up by antigen-presenting cells with the capacity to migrate to the draining lymph nodes and initiate an adaptive immune response. However, it has been found that during a natural infection, both the innate and adaptive responses to sporozoites are limited [4], possibly due to the small sporozoite inoculum (<100) [5], the ability of the sporozoites to invade liver cells within minutes after entering the bloodstream [6], and/or the lack of strong toll-like receptor (TLR) agonists, such as lipopolysaccharides (LPS), due to *Plasmodium*’s eukaryotic nature. In fact, there are usually no clinical signs or symptoms of *P. falciparum* infection for at least 10–12 days, as the parasite takes 6–7 days to complete development within a liver hepatocyte during the liver stage before being re-released into the blood [7–9].

Sporozoites carried to the liver from the bite site via the bloodstream actively invade hepatocytes, forming an invagination of the host cell’s plasma membrane to create a parasitophorous vacuole (PV) where they reside, isolated from the hepatocyte cytoplasm [10]. Within the hepatocyte, the sporozoite grows and replicates, producing a schizont containing thousands of merozoites over the course of 6–7 days [9]. From a schizont initiated by a single sporozoite, up to forty thousand merozoites can be released into the bloodstream when the hepatocyte finally ruptures [11]. Merozoites invade red blood cells (RBCs), not hepatocytes, and their surface proteome is distinct from the sporozoite, thereby evading any specific adaptive immune response generated against a sporozoite. Merozoite release marks the end of the pre- or exo-erythrocyte cycle and the beginning of the erythrocytic phase of the life cycle. Although, there is little evidence for the development of sterilizing protection against the pre-erythrocytic stages during natural parasite exposure [4], and thus is not the topic of this review, it has been an effective target for vaccine strategies [12,13], including the recent identification of neutralizing human monoclonal antibodies [14–16].

During RBC invasion, the merozoite again forms a parasitophorous vacuole where it resides and either replicates asexually or initiates sexual differentiation. One erythrocytic asexual replication cycle lasts 48 hours and produces 16–32 new merozoites [17]. After merozoite release by RBC rupture, the cycle continues until the parasites are cleared by the immune response or chemotherapy or the patient dies. This stage of the infection is accompanied by obvious clinical signs and symptoms and the resulting humoral immune response has been associated with protection against severe disease [18,19], however the specific target antigens remain unknown.

There is also a subpopulation of intraerythrocytic parasites that undergo sexual differentiation within the RBC instead of replicating asexually (Figure 1). The sexual stages, called gametocytes, are essential for malaria transmission via a mosquito. In the case of *P. falciparum*, sexual differentiation takes 10–12 days as a single merozoite develops into either a male or a female gametocyte. Once mature, gametocytes only survive for ~5 days if not taken up in a blood meal by a mosquito [20]. Their death and clearance has not been well studied, but in contrast to schizont rupture, are not associated with specific clinical signs or symptoms and will not be covered in this review. When taken up in a blood meal, conditions in the mosquito midgut stimulate gametogenesis, leading to fertilization and ultimately sporozoite production and transmission through the population (Figure 1). Antibodies generated against gametocyte/gamete antigens, including Pfs230, Pfs48/45 and Pfs47, have been shown to block mosquito transmission in the mosquito midgut [21], but a selective protective immune response targeted only against immature intraerythrocytic gametocytes in the human host has not been described [22–24].

## Blood-stage replication and antigen release

As RBCs lack MHC-I, they cannot present antigen to CD8 T cells and stimulate a cytotoxic response [2]. However, the parasite does actively transport antigens across the parasitophorous vacuole to the RBC surface where they can be recognized by B cells and induce antibody production [17,24]. As the parasite grows, the parasite-infected red blood cell (iRBC) becomes stiffer and misshapen, allowing recognition and clearance by the spleen. During this transition from ring to trophozoite (Figure 1), *P. falciparum* proteins exported to the RBC cell surface form protrusions on the RBC plasma membrane, called knobs, that act to concentrate the expression of *P. falciparum* erythrocyte membrane protein 1 (PfEMP1) [25–27]. Different PfEMP1 proteins can adhere to distinct host proteins, including scavenger receptor (CD36), intercellular adhesion molecule 1 (ICAM-1), endothelial protein C receptor (EPCR), and chondroitin sulfate A (CSA) that are expressed on endothelial cells [28,29], thus tethering the iRBC to the walls of the microvasculature and in pregnant women, the placenta [30,31]. This microvascular sequestration removes schizont stage *P. falciparum* parasites from the peripheral circulation, thus evading clearance by the spleen [30,32]. Endothelial cell binding can also alter the microvasculature causing pathology, as is seen in severe malaria [33]. However, exposure on the iRBC surface also makes PfEMP1 a potential target for inhibitory antibodies [29]. In response, the parasite genome has evolved to contain 60 different *var* genes encoding the PfEMP proteins, of which only one is expressed at any given time [34]. Most *var* genes are also extremely polymorphic, with an enormous number of alleles present in the population that can recombine to generate new alleles; and this polymorphism has limited its development as a vaccine candidate [31]. The distinct binding specificities of different PfEMP1s have been associated with different pathologies. For example, parasites that express the PfEMP1 gene encoded by *var2csa* bind CSA which covers the syncytiotrophoblast surface contributing to placental malaria [35–37], while PfEMP1 genes containing domains that bind EPCR are associated with severe malaria [33,38,39]. PfEMP1 that bind EPCR compete with protein C binding and block production of activated protein C (APC), which is an anti-coagulant protease that elicits cytoprotective and barrier strengthening signals in endothelial cells and has effects on thrombin signaling [33]. By interfering with the interaction of APC-EPCR, PfEMP1 not only adheres to epithelial cells, but also contributes to endothelial barrier dysfunction and defects in coagulation. Less is known about the impact of adhesion on the host response for the other PfEMP1 binding profiles, but this remains an active area of investigation [40–42].

The iRBC remains sequestered as the parasite grows, replicates and finally ruptures, releasing its contents into the bloodstream: 16–32 new merozoites and residue material including residual hemoglobin from the RBC cytoplasm, the digestive vacuole containing hemozoin crystals and phosphoantigens such as (E)-4-hydroxy-3- methyl-but-2-enyl diphosphate (HMB-PP), which is recognized by γδ T cells [43]. The released material stimulates the innate immune response, leading to the first clinical signs and symptoms and setting the stage for the development of an adaptive response.

## Blood-stage antigen recognition

**Figure 2.**
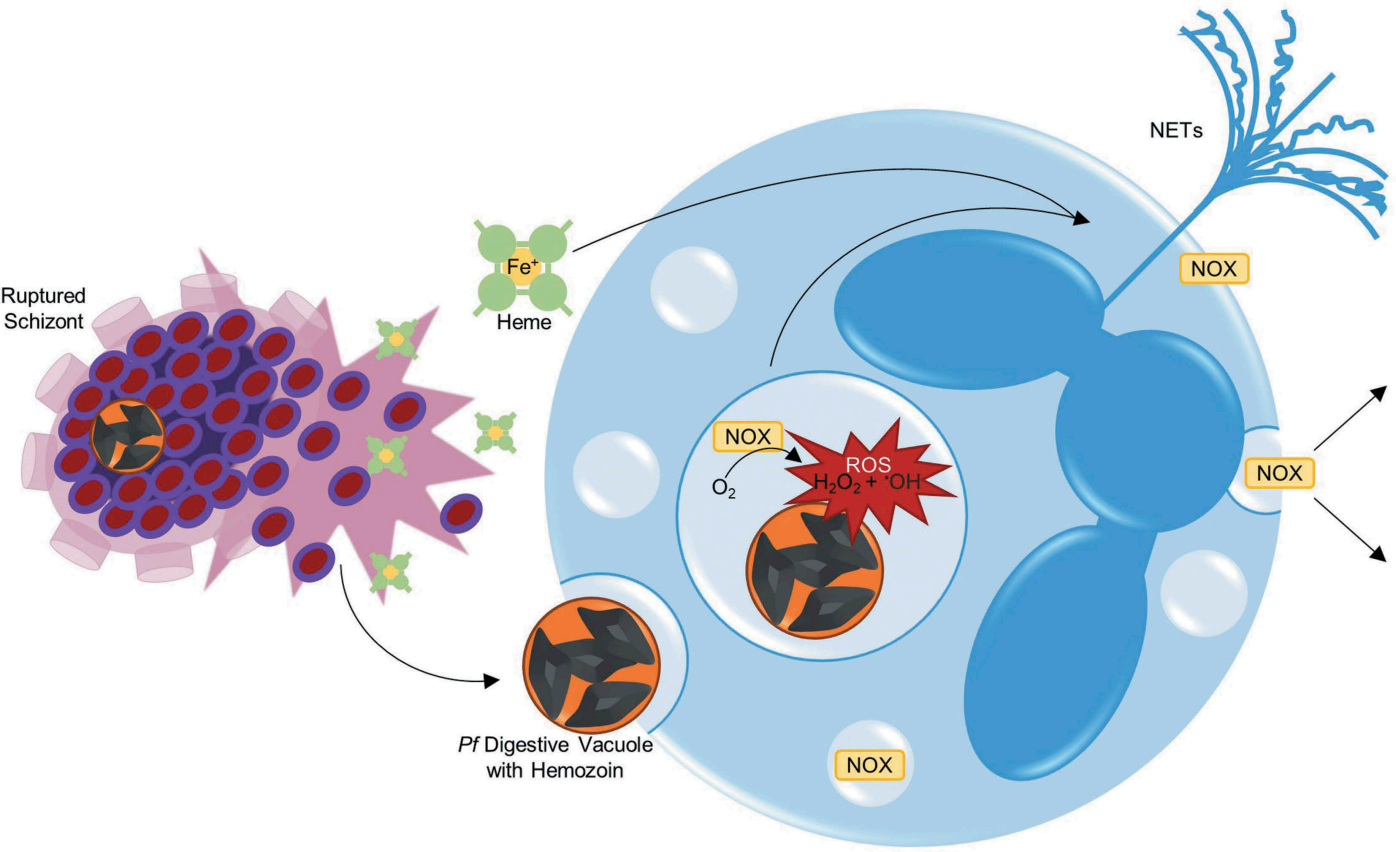
**Neutrophils engage in the innate immune response through phagocytosis and production of reactive oxygen species (ROS) and neutrophil extracellular traps (NETs)**. Following schizont rupture, antigenic debris are released from the iRBC, including heme and digestive vacuoles that contain hemozoin. Through their interaction with complement, neutrophils are able to phagocytose hemozoin-containing digestive vacuoles [82]. ROS occurs within this phagolysosome, a process by which oxygen is converted to superoxide by nicotinamide adenine dinucleotide phosphate (NADPH) oxidase (NOX), and then superoxide is converted into H_2_O_2_ and hydroxyl radicals (•OH). NOX can be present on the plasma membrane and phagosomal membrane, and is capable of diffusion outside of the neutrophil, as well [148]. Heme, a byproduct of RBC rupture during schizont release [88], can also trigger the release of NETs, structures composed of extracellular deoxyribonucleic acid (DNA), chromatin, and granule proteins that can ensnare and kill pathogens [80] .

**Figure 3.**
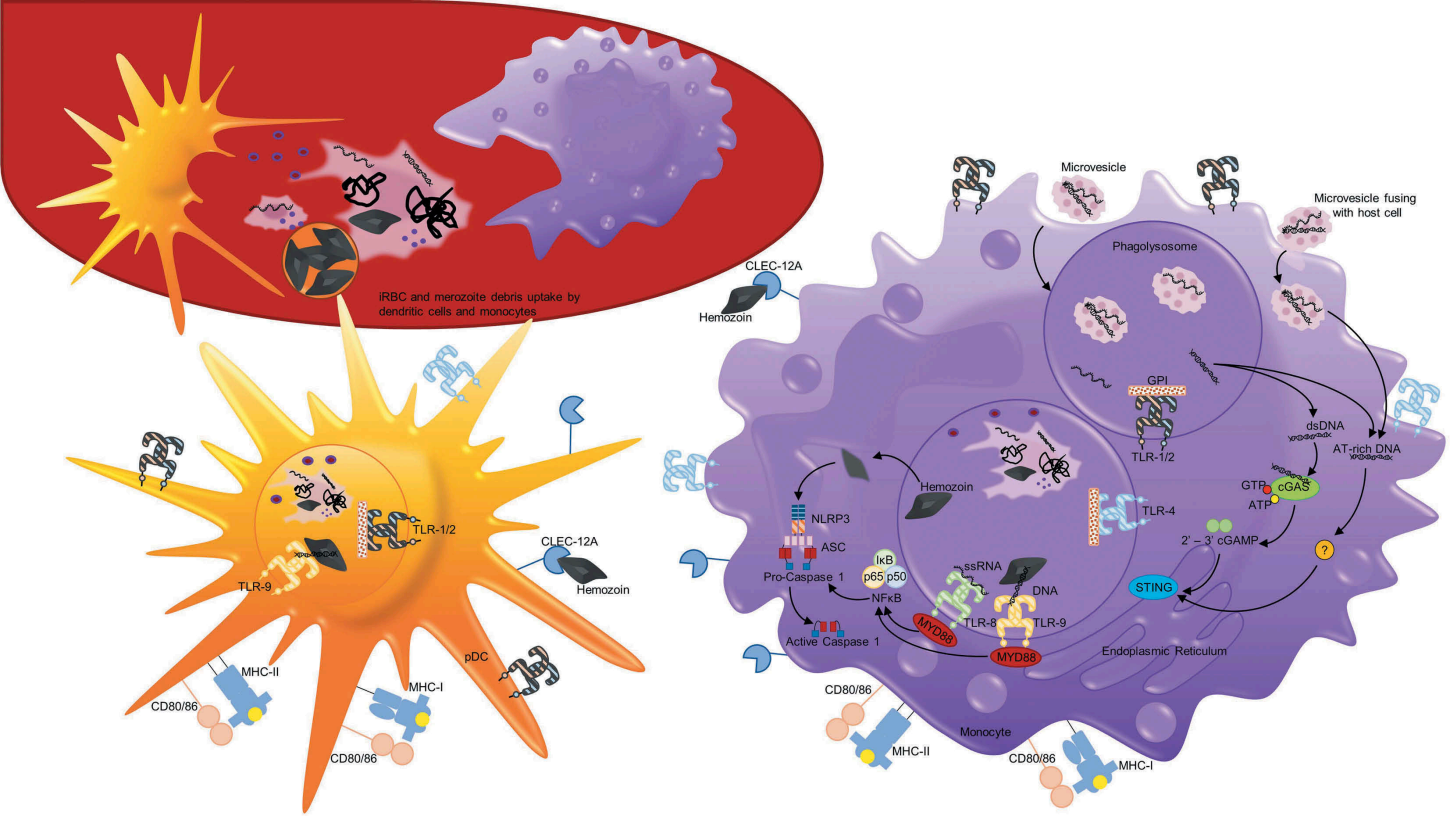
**Antigen uptake and recognition of *P. falciparum* during the erythrocytic cycle**. During each asexual cycle, schizont rupture releases merozoites as well as membrane fragments, DNA, RNA, and hemozoin that can be recognized and taken up by white blood cells, including dendritic cells and monocytes depicted here. *Plasmodium* antigens may also enter host cells, including monocytes, in the form of microvesicles that are phagocytosed or fuse with host cells. On their surface, monocytes and dendritic cells possess toll-like receptors including TLR1/2 and TLR4 that recognize glycophosphatidylinositol (GPI). They also possess C-type lectin receptor (CLEC) 12A that can specifically recognize extracellular hemozoin crystals. Additionally, after merozoites or ruptured parasite debris is phagocytosed, protein antigens can be degraded and presented on MHC molecules on the cell surface to activate T cells. Within the phagolysosomes, TLR8 can recognize ssRNA and TLR9 can recognize DNA that binds to hemozoin. TLR8 and TLR9 both signal through myeloid differentiation primary response protein 88 (MYD88), which activates nuclear factor-κB (NF-κB), and induces the production of proinflammatory cytokines via the activation of caspase 1. Hemozoin crystals can also destabilize the phagolysosomes, allowing entry into the host cytoplasm and activation of NOD-, LRR- and pyrin-domain-containing 3 (NLRP3) that assembles into inflammasomes. In the cytoplasm, NLRP3 combines with adaptor protein ASC to form Pro-caspase 1, and when activated, inflammasomes activate caspase 1 and cleave and secrete interleukin-1β (IL-1β). dsDNA that enters the cytoplasm with hemozoin or enters directly by microvesicle fusion to the plasma membrane can activate two additional signaling pathways. dsDNA can bind cyclic GMP-AMP synthase (cGAS), stimulating the generation of the second messenger cyclic GMP-AMP (cGAMP). 2ʹ-3ʹ cGAMP binds and activates stimulator of IFN genes protein (STING) in the endoplasmic reticulum that activates serine/threonine-protein kinase TBK1, leading to the production of type 1 interferons. AT-rich DNA in the cytoplasm can activate an unknown receptor that converges on this same STING–TBK1–IRF3 pathway.

While developing inside the iRBC, blood-stage *P. falciparum* parasites are protected from the innate immune response, including phagocytosis by neutrophils and monocytes, but after schizont rupture and merozoite release, parasite components are accessible to circulating white blood cells. γδ T cells, neutrophils, monocytes, dendritic cells and NK cells have all been shown to respond during these early stages of infection and these host-parasite interactions will be discussed in turn (Figures 2 and 3) [44,45]. The focus will be on the evaluation of the initial immune response in naïve individuals, which sets the stage for the development of long-term protection and can be studied directly using *in vitro* cultured *P. falciparum* parasites and controlled human malaria infections (CHMI). The analysis of the initial naïve response is not possible during a natural infection since often neither the timing of a patient’s most recent infection is known nor are the number of exposures that a patient may have experienced throughout their lifetime. The elucidation of the initial naïve response provides a baseline to compare with response to repeat infections to better understand the interplay between the innate and adaptive immune responses that lead to the development of protection against clinical disease that develops in endemic populations.

## Controlled human malaria infection (CHMI)

CHMI data from naïve subjects demonstrate that parasites are released into the blood 7 days after sporozoite introduction and symptoms usually begin when parasite levels reach >10 parasites/µL and can be detected by Giemsa-stained thick smear [7,8]. Following exposure to five *P. falciparum* infected mosquitoes, parasitemias usually reach this level between day 10–13, which is 3–6 days after release from the liver and corresponds to 2–3 cycles of asexual replication in the bloodstream [8]. CHMI subjects are normally treated when they become blood smear positive, which limits the parasite burden and disease progression, but provides a well-controlled platform to evaluate the early immune response. αβ T cell and B cell responses begin to be detected following treatment about 21–30 days after mosquito exposure [46–48].

## iRBC-induced cytokine profile *in vitro* and post-CHMI

*In vitro* stimulation of whole blood and PBMCs isolated from malaria naïve individuals is the most direct test for an innate immune response and demonstrates that high concentrations of *P. falciparum* schizont-stage parasites (2,000/µL) stimulate production of TNFα, IL-8, IL-1β, IL-6, IFNγ and GM-CSF within 6 hours [44,49]. Production of TNFα, IL-8, IL-1β, IL-6, plateaus at 9 hours, while IFNγ and GM-CSF continue to increase steadily for 24 hours. IL-10 levels do not increase until 12 hours and plateau at 18 hours. This rapid response is consistent with iRBCs stimulating an innate immune response. IFNγ plays a key role in the activation of monocytes and dendritic cells by enhancing phagocytosis, intracellular killing, cytokine secretion and antigen presentation [50]. Levels have been shown to be inversely correlated with total parasitemia during CHMI [51]. In contrast, IL-10 is considered an inhibitory cytokine that reduces expression of cytokines and antigen presentation, basically modulating the effects of IFNγ [50]. Consistent with this antagonistic role, IL-10 levels have been positively correlated with total parasitemia during CHMI [51]. TNFα, IL-1β and IL-6 act systemically as pyrogens and proinflammatory signals, increasing the body’s temperature set point, muscle metabolism and the production of acute phase proteins by the liver [52]. Both IFNγ and TNFα induce the production of nitric oxide and toxic radicals that can kill *P. falciparum* parasites [53]. In Gabonese patients, plasma levels of TNFα and nitric oxide were associated with parasite clearance and resolution of fever, although overproduction of either was associated with severe malaria [54–56].

Further *in vivo* CHMI studies reveal that blood-, not liver-, stage parasites induce the secretion of enough IFNγ, TNFα, IL-6, IL-10, IL-12p70, and B cell activating factor (BAFF) to be detected in plasma samples from volunteers within 4–8 days after parasite release from the liver [46,49]. Although levels vary between individuals, IFNγ is the predominant cytokine in the majority of subjects, which differs from *in vitro* data using high iRBC concentrations where TNFα and IL-1β predominate at early time points [49]. After exposure to *P. falciparum*-infected mosquitoes, IFNγ levels increase at least 20–82 fold over baseline levels during the 4–8 day blood-stage, and IL-10 levels usually follow the increase in IFNγ, but only reach 2.6–15.6 times baseline [49]. Interestingly, in a subpopulation of volunteers, TGF-β levels increase as parasites were released from the liver. This spike of TGF-β is associated with a decrease in the induction of the other cytokines and a slight increase in parasitemia [49], consistent with its role modulating proinflammatory immune responses [50].

Samples obtained from blood smear positive CHMI subjects just prior to treatment were also compared to symptomatic adult malaria patients living in malaria-endemic areas to evaluate the role of prior parasite exposure on the immune response [57,58]. Similar RNA profiles were obtained for both the CHMI subjects and symptomatic, malaria-experienced adults, with increases in IFNγ, TNFα, IL-1β, as well as the related nuclear factor kappa-light-chain-enhancer of activated B cells (NFκB) signaling and regulatory cascades [57]. The only major difference observed between the two groups was an increase in transcripts related to IL-10 and MAP kinase pathways in the symptomatic, malaria-experienced adults, which could both be involved in downregulating the immune response. IL-10 can directly inhibit the activation of macrophages, while MAP kinases can stimulate host cell apoptosis [57,59]. A similar strong IFNγ response was observed both in symptomatic malaria experienced adults and smear positive CHMI subjects using RNA-seq [58]. However, unexpectedly, no significant differences were observed between unexposed controls and naturally infected asymptomatic adults. These results suggest that asymptomatic, malaria-experienced adults did not respond to parasite levels ranging from 1000–8100 parasites/μL blood, which were ≥10 times higher than the parasite levels that induced a response in previously naïve CHMI subjects (range 4.1–100 parasites/µL blood). The underlying mechanism responsible for the reduced responsiveness of malaria experienced adults to blood-stage parasites remains unknown. At the single time point assayed there was no increase in inhibitory pathways, such as TGF-β, IL-10 or MAP kinase pathways in asymptomatic parasite carriers [58]. Future CHMI studies evaluating the immune response in adults with distinct malaria exposures are needed to extend these findings.

## Innate response

The cellular sources of the cytokines following iRBC stimulation have been evaluated and γδ T cells have the potential to be an early initial source of IFNγ and will be discussed first [60]. IFNγ released by γδ T cells would contribute to the activation of neutrophils, monocytes, macrophages and dendritic cells that have also been shown to recognize parasite materials. The resulting phagocytosis and secretion of additional cytokines are the first step toward the activation and antigen presentation needed to initiate an adaptive immune response. The secreted cytokines also activate NK cells inducing granulysin and IFNγ release, which contribute to parasite clearance and further stimulation of the innate and adaptive responses. The stimulation of each of these cell types by iRBCs will be discussed in turn to begin to understand the initiation of the immune response against *P. falciparum.*

## γδ T cells

γδ T cells, in contrast to αβ T cells, express highly restricted T cell receptors (TCRs) that can be divided into a small number of known subsets, all of which respond directly to non-peptide antigens, typically phospholipids [61]. The subset most commonly associated with malaria is γ9δ2 T cells, which are unique to humans and primates. Over 25 years ago, γ9δ2 T cells from patients in endemic countries and previously naïve patients in CHMI studies were shown to proliferate in response to *P. falciparum in vitro* [62–64]. In more recent studies of children in malaria endemic Papua, New Guinea, γδ T cells and CD14^+^ monocytes are the primary cellular sources of cytokines and chemokines associated with severe malaria [65]. γδ T cells comprise roughly 10% of PBMCs and up to 25% of hepatic T cells, providing immediate immunosurveillance [66,67] since they are not MHC-restricted and are thus able to respond to antigens without an antigen-presenting cell [68].

The γ9δ2 TCR recognizes the phospholipid (*E*)-4-hydroxy-3-methyl-but-2-enyl pyrophosphate (HMB-PP) which is produced primarily by prokaryotes that use the non-mevalonate pathway for isoprenoid synthesis. Unlike most eukaryotic cells, there was a secondary endosymbiotic event in the common ancestor of *Plasmodium* and other apicomplexan parasites that resulted in the formation of a relict plastid called an apicoplast [69] and the acquisition of genes from a number of prokaryotic pathways, including the non-mevalonate pathway for isoprenoid synthesis [70–73]. Residual HMB-PP released during schizont rupture can bind and activate host γδ T cells immediately [43,74]. Activated γδ T cells secrete cytokines, including IFNγ, that activate additional innate cells, and also release cytotoxic substances such as granulysin that can damage iRBC and merozoites preventing RBC invasion [60,75]. Additionally, activated γδ T cells can migrate to the lymph nodes where they act as antigen-presenting cells to initiate the adaptive immune response [43,76]. Following initial CHMI infection, γδ T cells have been shown to expand following infection and continue to be elevated for over a year [60,77].

## Neutrophils

Neutrophils, or polymorphonuclear (PMN) cells, are the most prevalent white blood cell and are often one of the earliest cell types that respond to infection during the innate phase through complement-dependent phagocytosis, formation of neutrophil extracellular traps (NETs), and production of antimicrobial products including reactive oxygen species (ROS) (Figure 2) [78–80]. Early *ex vivo* work showed that neutrophils from the blood of soldiers who returned from Vietnam with relapsed *P. falciparum* malaria could recognize and phagocytose parasite material released upon schizont rupture, but not intact iRBCs [81]. This observation was later extended to demonstrate that in the presence of serum from malaria naïve subjects, the released hemozoin-containing digestive vacuoles, not merozoites, were opsonized with complement, phagocytosed, and stimulated an oxidative burst (Figure 2) [82]. Complement inactivation by heating the malaria naïve serum reduced phagocytosis, which is consistent with complement-enhancing neutrophil recognition of parasite antigens as has been documented in a number of other studies [83,84]. Phagocytosis was further augmented by the addition of serum from malaria-exposed individuals in all these studies, suggesting that antibody-mediated opsonization could be an important effector mechanism to remove parasite material from the circulation following the induction of an adaptive immune response. Paradoxically, phagocytosis of *P. falciparum* digestive vacuoles appear to blunt subsequent ROS responses. This was demonstrated by Dasari *et al in vitro* when they co-cultured PMNs with digestive vacuoles containing hemozoin, and then challenged them with *Staphylococcus aureus*. Their experiment showed that after the initial burst of ROS following phagocytosis of *P. falciparum* digestive vacuoles, secondary ROS reactions upon the addition of *S. aureus* were significantly reduced compared to control PMNs cultured with media only [82]. Impaired oxidative responses were also observed when neutrophils isolated from malaria patients were exposed to an activation stimulus, phorbol 12-myristate 13-acetate (PMA), that stimulates protein kinase C [85,86]. Monocytes isolated from the same patients were not affected. The underlying mechanism was difficult to define in a small clinical study, but hypothesized to be related to increased plasma levels of heme, as has been found in a mouse malaria model [87]. Heme is released during hemoglobin digestion and consequently is a biproduct of RBC lysis during schizont rupture. Free heme itself, independent of malaria infection, is highly cytotoxic, promoting oxidative stress that can cause endothelial injury [86]. Heme, but not the intact iRBC, was found to potently stimulate NETosis in TNF-primed neutrophils (Figure 2) [88]. The release of NETs, structures composed of extracellular deoxyribonucleic acid (DNA), chromatin, and granule proteins, by activated neutrophils can also ensnare and kill pathogens [80] and have been implicated in malaria pathogenesis (Figure 2) [88,89]. The heme levels required for stimulation (>20 µM) were consistent with heme concentrations found in plasma during malaria infections [88]. Additional stimuli for NETosis have been proposed, including uric acid crystals, hypoxanthine, and extracellular hemozoin that are released following iRBC rupture [88,90,91], cytokines like TNFα and IL-8 [92], and hydrogen peroxide (H_2_O_2_) that is produced during ROS (Figure 2) [90,93].

Further work is needed to integrate these intriguing *in vitro* and *ex vivo* findings with the responses of other white blood cells, as well as the lack of neutrophil accumulation at sites of pathology during severe malaria. However, a number of challenges limit the study of neutrophils in malaria, as well as in other diseases. The first challenge is that neutrophils are a highly sensitive cell population that cannot be propagated in culture for more than a day and cannot be cryopreserved, so all analysis has to be performed quickly on freshly isolated cells. Neutrophils are also not co-isolated during standard PBMCs preparations, therefore the only human neutrophil data has come from counts of general clinical blood collections, even during CHMI studies. For example, van Wolfswinkel *et al* used clinical blood collection data to find that after naïve subjects were exposed to *P. falciparum* injected mosquitoes, peripheral blood neutrophil numbers were stable during the liver-stage of infection, but then decreased to borderline neutropenia during blood-stage infection [94]. Whether the reduction in circulating neutrophils was due to clearance, NETosis, migration to sites of parasite sequestration, or some combination of these remains unknown. As mentioned above, there is no evidence for an accumulation of intact neutrophils at sites of parasite sequestration, but further research is needed to evaluate the presence of NETs [88]. Future CHMI studies should include collections that enable neutrophil analysis, including rapid staining for flow cytometric analysis and modifying WBC isolation procedures to collect neutrophils and PBMC separately.

## Monocytes

Monocytes make up 10–30% of PBMCs and express complement receptors and a range of pattern recognition receptors (PRR) that allow a rapid response to pathogens, including phagocytosis and cytokine/chemokine release [95]. Monocytes can also migrate into tissues and differentiate into macrophages or myeloid dendritic cells. *Plasmodium* DNA, RNA, glycosylphosphatidylinositol (GPI) anchors and hemozoin crystals have all been found to activate monocytes, macrophages and dendritic cells (Figure 3) [96]. All these antigens are released from the iRBC when schizont-infected RBCs rupture, while ring stage iRBCs release microvesicles containing *P. falciparum* RNA, DNA and proteins that have been shown to be taken up by surrounding cells (Figure 3) [97,98].

Early work demonstrated a response against *P. falciparum* GPI anchors, which function as the membrane attachment site for many of the *P. falciparum* surface proteins. The malarial GPI is degraded by macrophage surface phospholipase A_2_ and phospholipase D and then recognized by TLR2 and TLR4 (Figure 3) [99]. Preferential activation of TLR1/2 pairs by GPI indicates that the lipid portions of *P. falciparum* GPI ligand are critical for TLR recognition [100]. Research on host recognition has found that *P. falciparum* GPI is also recognized by CD1d-restricted NKT cells [101]. In contrast to *in vitro* results and co-culture experiments, human CHMI studies have failed to indicate that NKT recognition of *P. falciparum* antigens is as prevalent during early stages of infection [102–104]. However, during CHMI lymphopenia is commonly observed after parasites are released from the liver, which includes a reduction in NKT cells. It has been suggested that this decrease in circulating lymphocytes corresponds with their retention and potential activation in the lymphoid tissue by antigen-presenting dendritic cells.

An innate immune response against hemozoin purified from lysed parasites has also been demonstrated [105–109]. Hemozoin is an inert crystal produced in the food vacuole from the heme extracted during the digestion of host RBC hemoglobin. It is released during schizont rupture and is readily phagocytosed by monocytes, macrophages and dendritic cells, (Figure 3) [106] in addition to neutrophils which have already been discussed. Initial work suggested that the phaogocytosis of hemozoin causes monocytes to undergo oxidative burst and downregulate MHC-II, ICAM-1, and CD11c expression [105,106]. However, iRBC or hemozoin has also been shown to stimulate B cell activation factor (BAFF) secretion by monocytes, which could enhance antibody production [109]. Later, it was found that hemozoin stimulated type 1 IFN production in PBMCs [107], but on further analysis the stimulus was found to be *Pf*DNA associated the hemozoin that bound to host TLRs [108]. *Pf*DNA introduced into the endolysosomal compartment of Fms-related tyrosine kinase 3 ligand (FLT3-L) stimulated murine bone marrow cells was recognized by TLR9 leading to type 1 IFN production [110] through the activation of NFκB, while introduction into the cytosol allowed *Pf*DNA to bind to cyclic GMP–AMP synthase, stimulating the STING pathway which results in type 1 IFN production and leads to the production of TNFα and IL-6 [108,111]. It has also been reported that once in the cytoplasm, the hemozoin crystal itself activates the cytosolic NLRP3 inflammasome, leading to the activation of caspase 1 and secretion of IL-1β [96,112–114] (Figure 3). Just this year, hemozoin alone has been shown to be recognized directly by the C-type lectin receptor (CLR) CLEC12A on the surface of granulocytes and phagocytic cells, including dendritic cells and macrophages [115]. Once phagocytosed, parasite-produced hemozoin, which can reach levels of 0.2–2.0 g in the bodies of malaria-infected patients with a parasitemia of 1–10% [116], enters and then disrupts the endolysosomal pathway, allowing access to the cytoplasm [96,108,111]. It has also been reported that *Pf*DNA, RNA and protein containing microvesicles released into the circulation from RBCs infected with ring stage parasites [97,98] can be taken up by monocytes and the contents released into the cytoplasm [42,117]. Once in the cytoplasm, *Pf*DNA again was shown to activate the STING pathway, leading to the production of type 1 IFN as well as chemokines CCL5 and CXCL10 [117]. Interestingly, microvesicles lacking PfEMP1 did not alter the type 1 IFN response, but induced more robust changes in the transcriptome of the target cells, suggesting PfEMP1 may act to downregulate the innate response [42]. The same research group also found that PfEMP1-null parasites increased NFκB transcription factor activation in a macrophage cell line 2-fold compared to PfEMP^+^ parasites, resulting in increased TNF and IL-10 release, while primary human monocytes released 2 times more IL-1β, IL-6, IL-10, monocyte chemoattractant protein 1 (MCP1), macrophage inflammatory protein 1α (MIP1α/CCL3), MIP1β/CCL4, and TNFα [41,42]. These results are consistent with a role for PfEMP1 in downmodulating the innate response, but the actual mechanism remains to be determined. *Pf*RNA has also been shown to bind endosomal TLR8, not TLR7, in human monocytes and stimulate the secretion of IL-18 and IL-12p70, which in turn stimulate the release of IFNγ from NK cells [118].

## NK cells

NK cells were one of the earliest cell types identified to be associated with malaria protection, as NK cells were found to be correlated with high titers of interferon and parasitemia in a study of endemic children [119]. In a humanized mouse model, human NK cells were able to clear *P. falciparum* [120]. However as indicated above, NK cells depend upon multiple, contact-dependent and cytokine-mediated signals from monocytes and myeloid/conventional dendritic cells (cDCs) to produce IFNγ in response to iRBCs [121]. McCall *et al* also found that *in vitro* when NKT and T cells were depleted, but not γδ T cells, NK cells were unable to produce IFNγ in response to iRBCs. Conversely, NK cells were not necessary for T cells to produce IFNγ in response to iRBCs [75]. However, once stimulated, NK cells can then augment the response by activating resting cDCs and monocytes [121].

Recently, NK cells have also been found to be activated by *Plasmodium* RNA contained within microvesicles via the MDA5 pathway [122]. NK cells are important members of the innate immune response as they comprise up to 13% of blood lymphocytes and, upon activation, can secrete chemokines, cytokines such as IFNγ, and cytotoxins that contribute to iRBC lysis [123,124]. Following their activation and upregulation of CD25, NK cells are able to release cytokines in response to local and systemic inflammation [125–127]. It has been suggested that individuals differ in their ability to initiate an innate, NK cell-mediated response due to differences in their ability to ability to upregulate CD25 and LAMP-1 [125], which contribute to variation in individuals responses to *Plasmodium* parasites. CD25 reflects an NK cell’s proliferative capacity and LAMP-1 is indicative of an NK cell’s functional activation, as expression is associated with cytotoxic granule exocytosis.

In malaria naïve subjects prior to CHMI, NK cells made up 14% of the IFNγ^+^ lymphocytes and this did not change during the early stages of infection. Starting about day 35 post-infection, or 3 weeks after treatment, NK cell levels increased and remain elevated until day 140 (20 weeks) post-infection, mirroring increases in CD3^+^ T cell populations [75]. By this time post-infection, as more T cells began expressing IFNγ, only 7% of the IFNγ^+^ lymphocytes were NK cells [75]. In contrast, Mpina *et al* found that during a CHMI performed in a malaria endemic region of Tanzania using aseptic *P. falciparum* sporozoites (*Pf*SPZ challenge), subjects experienced significantly reduced NK cell frequencies in the blood at 28 and 56 days post-challenge [103]. This response also appeared to be dose-dependent as these results were particularly apparent in patients in the high challenge cohort [103]. Further *in vitro* studies evaluating the PBMCs isolated from subjects that grew up in malaria endemic areas will be needed to understand the differences and determine whether they contribute to the protection against clinical malaria [128].

## Dendritic cells

Although dendritic cells only comprise ~1% of peripheral blood lymphocytes, they serve vital functions as they not only respond to early signs of infection by recognizing pathogen-associated molecular patterns (PAMPs), but can also load and present antigen complexed with MHCI or II for presentation to T cells. Once stimulated, dendritic cells initiate the adaptive immune response by migrating to draining lymph nodes where they present antigen/MHCI or II complexes, as well as costimulatory signals including CD40, CD80, and CD86, to CD4^+^ and CD8^+^ T cells (Figure 3) [129]. Naïve CD4^+^ and CD8^+^ T cells that recognize the antigen/MHCII or MHCI complexes, respectively, with the appropriate co-stimuli proliferate and differentiate into effector cells.

Dendritic cells had been divided into 2 main categories, plasmacytoid [CD123^+^CD11c^−^, (pDC)] and myeloid/conventional [CD123^−^CD11c^+^ (cDC)] [130]. While pDCs are typically associated with protection during viral infections, they express high levels of TLR7 and TLR9 and can be an important source of Type 1 IFNs during other infections [130,131]. cDCs can be derived from monocytes and become efficient antigen-presenting cells once maturation markers are upregulated following activation [132]. Detailed transcriptomic profiling has expanded the classifications for the cDCs [131,133]. This recent work provides insight into how distinct DC populations can induce specific adaptive responses, but complicates the interpretation of prior work characterizing the responses of DCs as a whole. Both cDC and pDC have receptors for different ligands, including *Plasmodium* byproducts, and have been evaluated *in vitro* and following CHMI.

Human DCs avidly phagocytose *P. falciparum* infected RBCs *in vitro*, but activation is dose- dependent. A high ratio of 100 iRBC:dendritic cell (iRBC:DC) actually inhibits maturation, inducing apoptosis and blocking stimulation by LPS [134,135]. In contrast, at a 3:1 iRBC:DC ratio maturation markers, HLA-DR, CD80, CD86 and CD40, and chemokine, CCL2, CXCL9 and CXCL10, expression increase, although no cytokines are produced [136]. A similar profile, with an increase in maturation marker expression without an increase in cytokine expression, was also observed when DCs from adults living in a malaria endemic area were tested [136]. However, when only CD16^+^CD1c^−^ cDCs were examined, an increase in intracellular expression of TNF and IL-10 were observed in response to iRBCs [137]. At a 1:3 ratio of iRBCs, both DCs and isolated cDCs activated an antigen-specific T helper 1 (Th1) cell response so that restimulation after 11 days resulted in T cell proliferation and secretion of IFNγ, IL-10 and TNFα.

The role of pDCs is less clear, although when stimulated with iRBCs in the presence of cDCs, secretion of Type 1 IFN, CXCL9 and CXCL10 was increased. CXCL9 and CXCL10 play a role in the induction of type I regulatory T cells (Tr1), which are increased during malaria [138]. Although, type 1 IFNs bind to receptors on many cells, stimulating anti-viral defense strategies [139], they also have been shown to inhibit parasitic-specific CD4^+^ T cell IFNγ production and promote parasitic-specific IL-10-producing Th1 or Tr1 cells [51,140,141]. Type 1 IFNs also reduce the ability of blood monocytes to produce inflammatory responses by inhibiting IL-6 production [51].

pDC and distinct cDC subpopulations have also been assessed *in vivo* following CHMI in naïve adults. Only CD16^+^ cDCs were found to increase expression of maturation markers, HLA-DR and CD86, following infections initiated with either sporozoites [142] or iRBCs [137]. CD16^+^ DCs isolated at peak parasitemia also had increased functionality during *in vitro* restimulation with iRBCs, with increased TNF production by both TNF-only and TNF/IL-10 producing CD16^+^ DCs [137]. In response to *in vitro* TLR ligation, CD16^+^ DCs isolated during peak parasitemia produced increased IL-10 and IL-12 [137]. In contrast, maturation marker expression did not increase on either CD1c or BDCA3 cDCs or pDCs [142–144] and levels of circulating pDCs and CD1c^+^ cDCs both decreased following infection with a dose of 1,800 iRBCs. There was also a concomitant increase in the percent of DCs staining with the apoptosis markers, annexin V [145] and active caspase 3 [143,144]. At peak parasitemia, circulating pDCs were still functional *in vitro*, producing IFNα and TNF and upregulating CD86, HLA-DR, and CD123 following TLR7 and TLR9 stimulation [144], while CD1c^+^ cDCs expressed lower levels of HLA-DR, which correlated with less phagocytic capacity as well as impaired signaling following TLR or iRBC stimulation [143]. iRBC- and TLR1/2 and TLR4 agonist-induced CD86 expression was also lower in CD1c^+^ cells isolated at peak parasitemia than prior to infection, while there was no change in TLR1/2 and TLR4-induced IL-12 expression and all three stimuli, iRBCs, TLR1/2 and TLR4 agonists, increased intracellular TNF [143].

Together the results are consistent with *P. falciparum* infection having differential effects on distinct populations of circulating DCs, with *in vivo* iRBC stimulation during the initial infection activating CD16^+^ DCs and inhibiting circulating CD1c^+^ cDCs, while pDCs are not affected. The specific stimuli have not been identified, but previous work has demonstrated that CD16^+^ DCs and CD1c^+^ DCs express similar TLRs other than TLR3, which is not expressed by CD16^+^ DCs. CD16^+^ DCs were also found to secrete higher levels of cytokines than CD1c^+^ DCs, except for IL-8, and to more potently activate endothelial cells [146]. It is more difficult to study the population of DCs that leave the circulation and migrate to the lymph nodes to initiate the adaptive response, but this efflux could influence the composition of the circulating DCs. Lymphopenia is observed during CHMI, suggesting antigen-specific T and B cells are being retained and activated in the lymph nodes, but biopsy material would be needed to characterize this important cell population [132,143].

Given the ability of pDCs to recognize *Pf*RNA and DNA and present antigen effectively, it is unclear why they remain unaffected during CHMI [130,131,144]. It is possible that they play a role later at the high levels of parasitemia and symptoms that are only attained during a natural infection, not a CHMI. Their ability to secrete high levels of type 1 IFNs that have been shown to inhibit the innate and adaptive responses could serve to downregulate the immune response at high parasitemia. More work is needed to identify the stimuli and signaling pathways induced during *P. falciparum* infections to better understand the interactions between the innate response, as well as the impact on the development of protective immunity.

## Conclusion

*In vitro P. falciparum* iRBC components are recognized by malaria-naïve γδ T cells, neutrophils, monocytes and dendritic cells, initiating an innate immune response. Specifically, parasite antigens, including HMB-PP, can stimulate γδ T cells to secrete IFNγ, TNF, MIP-1α/CCL3 and MIP-1β/CCL4 [65], which can activate neutrophils, monocytes and dendritic cells, thereby augmenting phagocytosis, as well as antigen presentation, and the secretion of inflammatory cytokines and chemokines by monocytes and dendritic cells. The secretion of IL-12p70 and IL-18 by monocytes in response to *Pf*RNA also leads to the activation of NK cells a major source of IFNγ secretion during malaria infections that potentiates the activation of the immune response [118]. The innate response is also capable of sufficient antigen presentation and co-stimulation to initiate an adaptive response by activating CD4^+^ T cells that secrete IFNγ and TNFα in response to *P. falciparum* antigens [147].

*In vivo* during a CHMI, the innate response is more variable. The majority of naïve subjects respond to blood-stage infection with the secretion of IFNγ by γδ T cells and NK cells, as well as αβ T cells and levels of IFNγ continue to increase for a week after treatment [51,65]. Just after IFNγ levels begin to rise, different subjects expressed different levels of TNFα, IL-6, IL-10, IL-12p70, and BAFF production [46,49]. The reason for this variation remains unclear due to the limited number of cell types analyzed for cytokine expression. Recent advances, such as single cell sequencing [131], should allow additional discrimination of the transcription profiles of the cells associated with these individualized responses to parasites. The use of CHMI to compare the responses of malaria-naïve and experienced subjects over the course of the infection should also shed light on specific responses that correlate with clinical protection and allow the design of strategies to reproduce these protective responses in naïve individuals.
